# An Investigation on Water Quality of Darlik Dam Drinking Water using Satellite Images

**DOI:** 10.1100/tsw.2010.125

**Published:** 2010-07-06

**Authors:** Erhan Alparslan, H. Gonca Coskun, Uğur Alganci

**Affiliations:** ^1^ Marmara Research Center, Earth and Marine Sciences Institute, TUBITAK, 41470 Gebze, Kocaeli, Turkey; ^2^ Department of Remote Sensing, Faculty of Civil Engineering, Istanbul Technical University, 34469 Maslak, Istanbul, Turkey; ^3^ Center for Satellite Communication and Remote Sensing, Istanbul Technical University, 34469 Maslak, Istanbul, Turkey

**Keywords:** water pollution, remote sensing, geographic information systems, multiregression analysis, pattern recognition, watershed management

## Abstract

Darlik Dam supplies 15% of the water demand of Istanbul Metropolitan City of Turkey. Water quality (WQ) in the Darlik Dam was investigated from Landsat 5 TM satellite images of the years 2004, 2005, and 2006 in order to determine land use/land cover changes in the watershed of the dam that may deteriorate its WQ. The images were geometrically and atmospherically corrected for WQ analysis. Next, an investigation was made by multiple regression analysis between the unitless planetary reflectance values of the first four bands of the June 2005 Landsat TM image of the dam and WQ parameters, such as chlorophyll-a, total dissolved matter, turbidity, total phosphorous, and total nitrogen, measured at satellite image acquisition time at seven stations in the dam. Finally, WQ in the dam was studied from satellite images of the years 2004, 2005, and 2006 by pattern recognition techniques in order to determine possible water pollution in the dam. This study was compared to a previous study done by the authors in the Küçükçekmece water reservoir, also in Istanbul City.

## INTRODUCTION

With its population of over 12 million, according to the demography world news (DWN) of 30 November 2007, Istanbul is certainly one of the most populated cities of the world. This is because much of the population, about 400,000 people per year, has migrated here from various regions of Turkey, as almost half of the industry of the entire country is located in Istanbul City and its vicinity[2]. The annual population increase in the city is approximately 4.6%, which is twice the average of the country. This creates an annually increasing demand for a potable water supply. The annual water amount supplied to Istanbul City, bisecting two continents (Asia and Europe), was 709 million $m^3$ in 2004, 697 million $m^3$ in 2005, and 732 million $m^3$ in 2006, which was supplied from eight main reservoirs as shown in Fig. 1. One of these reservoirs is Küçükçekmece Lake, situated at sea level and separated from the sea by a shallow prominence formed by the sand and gravel swept away from the coast. The water quality (WQ) of the Küçükçekmece

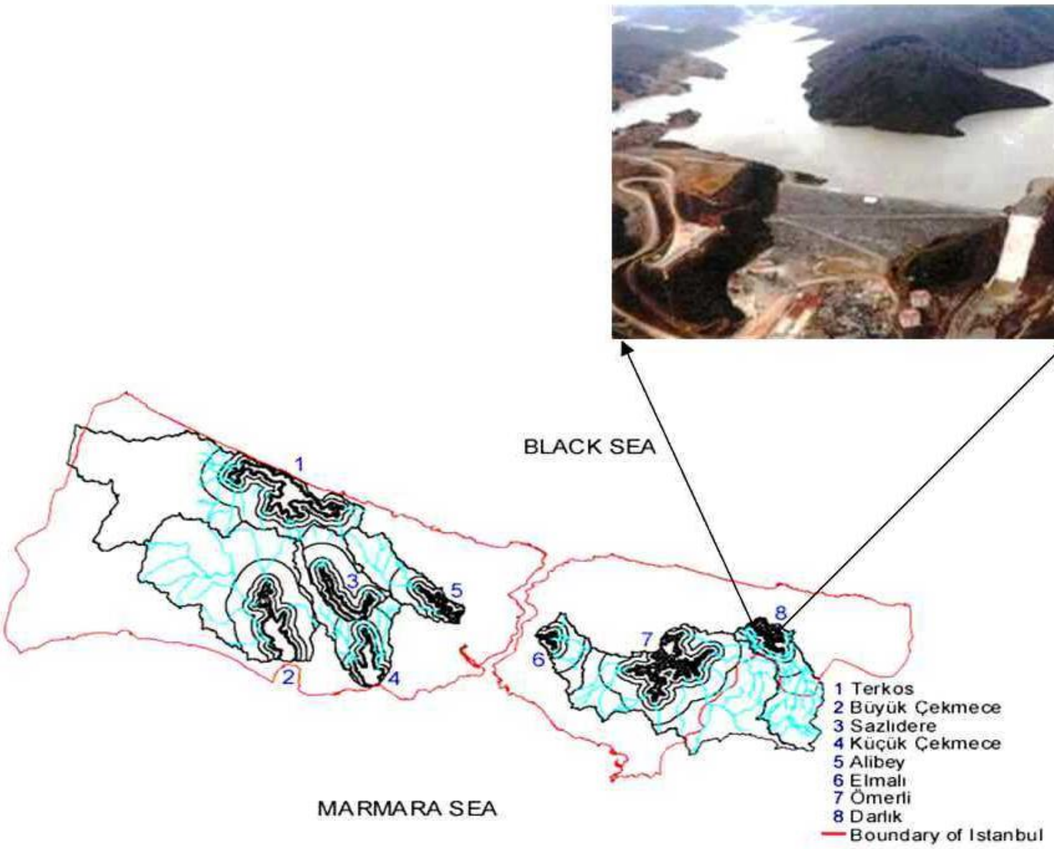

**FIGURE 1.** Location of Darlık and other reservoirs and their watersheds.

Lake was studied previously by the authors[1]. In contrast to Küçükçekmece Lake, Darlık Lake is a man-made dam lake, which was constructed on the Darlık Creek between the years 1986 to 1988 for domestic and industrial water supply purposes. The two water reservoirs display different watershed characteristics, mainly due to their geographic location; the current one being inland, while the previous is almost at sea level.

Water resources are vital for the well-being of a city. It is difficult to maintain WQ at an acceptable level for the potable use of the water supply or to maintain its sustainability. With an increasing population, WQ in the reservoirs therefore needs to be monitored continuously in order to take measures, when necessary, to sustain the potability of the water supply resources. Although field measurements or laboratory analyses provide accurate information for a known time and limited locations on potable water resources, the study is expensive and incapable of monitoring and evaluating regional WQ at definite periods when numerous sampling locations need to be selected for very large areas. Temporal land cover changes in the vicinity of water resources, particularly in their catchment areas, also need to be investigated to determine point or nonpoint sources of pollution in order to prevent deterioration of the WQ in water resources.

Over the past 3 decades, there have been significant advances in the development of space-based tools that offer rapid, repeated, and concurrent synoptic assessment of environmental parameters in oceanic and adjacent land areas. The academic research community has successfully demonstrated the applicability of satellite sensors to elucidate a variety of ecological processes. In the referred studies, reliable empirical relationships were developed between Landsat satellite data (http://landsat.gsfc.nasa.gov) and ground measurements of WQ[1,3,4,5,6].

The satellite remote sensing (RS) technique allows us to obtain reliable data for accurate WQ interpretation because satellite image data sensed by the optical and thermal sensors on various RS

platforms may be complemented with *in situ* measurements taken at or near satellite image acquisition time. These measurements help us to carry out multiple regression analyses in order to compute various WQ parameters on the entire water surface that will assist provincial administrators and city planners in the management of water resources. RS technology offers an economical and advantageous alternative over conventional WQ monitoring techniques in large areas at definite time periods[7,8,9].

The basis for WQ assessment by RS is spectral signatures produced by inorganic and organic substances within the water body that affect absorption, scattering, and downward refraction characteristics of the solar energy incident on the water surface. Unlike spectral reflection, the amounts of absorption and backscatter from a water body are highly dependent on the wavelength intervals (channels) being sensed by the recording instruments. Reflectance and backscattering are not only a function of the properties of the present sediments, but are also dependent, for example, on biological constituents such as phytoplankton. Distinct spectral signatures obtained from satellite observations correspond to water bodies of different composition. Therefore, remotely sensed satellite data must always be combined with ground truth (*in situ*) measurements in order to allow for an accurate and unambiguous interpretation[10,11,12].

## STUDY AREA

Darlik Dam is situated on the Anatolian side of Istanbul City, about 7 km south of Şile County. It has a rock-filled body of 1,600,000 $m^3$ volume and a riverbed altitude of 73 m. The reservoir volume and the lake area at normal water surface elevation are, respectively, 107 $hm^3$ and 6 $km^2$ http://wikimapia.org/9469197/tr/Darl%C4%B1k-Baraj%C4%B1

The study area of the Darlik Water Basin and Dam is presented in Fig. 2 as geo-referenced vector data with the protection zone defined around the lake according to the 1988 Water Pollution Control regulation of the Turkish Republic[13].

The WQ and water supply maintenance at Darlık Dam is under the control of the Istanbul Water and Sewerage Administration (abbreviated as ISKI in Turkish), which created regulations for establishment of four types of protected areas around Darlık Dam, as in other water resources of the city. These are called absolute protection zone (0–300 m), short-range protection zone (300–1000 m), medium-range protection zone (1000–2000 m), and long-range protection zone (2000 m to the water basin border)[14]. Statistical information on the Darlık water reservoir is given in Table 1[15]. According to Table 1, very few settlements are in the long-range protection zone, occupying only 1% of the Darlık Dam watershed area and there is no industry at all. This makes the WQ at Darlık Dam in the transition stage from high-quality water to slightly polluted water, which allows its utilization for water supply purposes after prescribed treatment. The current study investigates if there have been any changes in the WQ of Darlık Dam in the time period from 2004 to 2006 using RS, GIS, pattern recognition, and multiple regression analysis techniques.

## METHODOLOGY

The methodology followed is similar to that used for Küçükçemece Lake[2] and it consists of the following steps:

1. Investigation of multitemporal land cover changes in the protection zones of the Darlık Dam using Landsat 5 TM satellite images of December 2004, June 2005, and May 2006
2. Investigation of WQ at Darlık Dam in June 2005 by multiple regression analysis
3. Investigation of multitemporal changes in the Darlık Dam water resource using satellite images

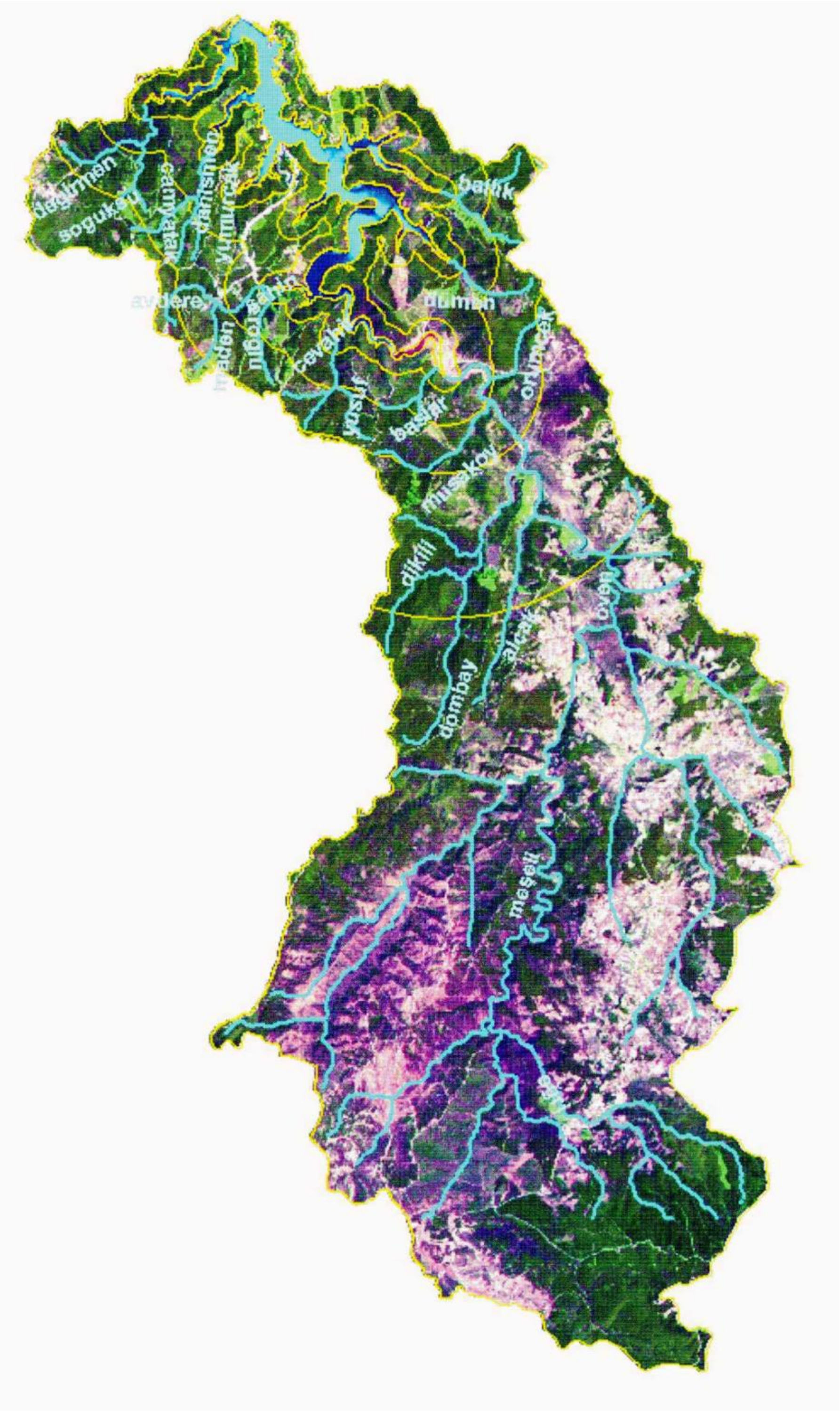

**FIGURE 2.** Rivers overlaid on Darlık watershed Landsat 5 TM satellite image.

**TABLE 1**
**Statistical Information on Darlik Dam[15]**

**Share of Darlık for water supply in Istanbul**

| Annual water production (millions $m^3$) | Share (%) |
|---|---|
| 97 | 15.9 |

**Area and settlement characteristics of Darlık Dam**

| Total area ($km^2$) | Lake area ($km^2$) | Protection Zone | Protection Area ($km^2$) | Protection % | Settlements (n) | Population N | Population % | Population Density (persons/$km^2$) | Industries (n) |
|---|---|---|---|---|---|---|---|---|---|
| 199 | 6 | Absolute | 11 | 6 | — | — | — | 0 | — |
| | | Short | 17 | 9 | — | — | — | 0 | — |
| | | Medium | 16 | 8 | — | — | — | 0 | — |
| | | Long | 149 | 77 | 7 | 1885 | 100 | 13 | — |
| | | Total | 193 | 100 | 7 | 1885 | 100 | 9 overall | — |

**Distribution of land uses in watershed of Darlık drinking water reservoirs**

| Forests/bush | Agriculture/meadows | Settlement/industry | Lake | Total area ($km^2$) |
|---|---|---|---|---|
| 72% | 25% | 1% | 3% | 199 |

**Classification of Darlık according to Water Pollution Control Regulation of Turkey**

| Physical and inorganic chemical parameters | Organic parameters | Inorganic pollution parameters | Bacteriological parameters |
|---|---|---|---|
| 2 | 2 | 2 | 1 |

**Classification of Darlık according to technical standards**

| Hydrographic or territorial criteria | Trophic criteria | Salinity, special criteria, and hygienically relevant criteria | Overall evaluation |
|---|---|---|---|
| 1 | Insufficient data | 2 | 2 |

A new technology rather than the one used in Tanik et al.[2] was not sought because RS and GIS technologies are novel technologies that proved themselves to be successful over conventional technologies in WQ assessment studies and these are therefore widely employed[16,17,18,19,20,21,22]. Our current study also showed this fact as elaborated below.

## Multitemporal Land Cover Change Detection

Urban growth in big cities can be objectively monitored using the information extracted from multitemporal satellite imagery[23,24,25]. In this study, multitemporal Landsat 5 TM satellite images of the Darlık Dam and its watershed acquired in years 2004, 2005, and 2006 were employed to investigate multitemporal changes. The main steps followed in the analysis were extraction of the study area from image frame, geometric correction, supervised image classification, and, finally, accuracy assessment. The details for these steps are given below.

The study area was first extracted from its image frame using ERDAS Imagine 8.7 image processing software.

Both 1:25,000- and 1:5,000-scaled digital maps that cover the study area and orthophotos derived from 1:5000 air photos were used as ground truth information for geometric correction. Each image was displayed on the computer screen and the map of the respective area having UTM coordinates was spread near the screen. For each image, 100 control points were homogeneously selected, both on the image and on the map at road junctions, lake coastal zones, and the geographical features that do not change in time.

Using these control points, the raw satellite image subframes hosting the study area were warped and projected into UTM coordinates by linear interpolation with, at most, 0.1 RMS error.

Registered satellite images were then classified into 80 classes by the supervised maximum likelihood (Bayesian) decision rule within the ERDAS Imagine 8.7 software. These 80 classes were then reduced in number to ten final classes by aggregation. These ten classes were verified on the computer display screen by confirmation through the ground samples taken from the experimental area with accurate field survey information near the satellite image acquisition date.

Accuracy assessment was made for the classified images of the years 2004, 2005, and 2006 by choosing 100 random pixels and comparing the results with fieldwork. The accuracy achieved in classification were 84, 86, and 87%, respectively, for the years 2004, 2005, and 2006 for the randomly selected 100 points. Urban areas detected in absolute, short-range, medium-range, and long-range protection zones of the Darlık watershed are tabulated both in area and as occupation percentage of the watershed area for the years 2004, 2005, and 2006 in Table 2. Table 2 also shows the annual and biennial changes in percent occupancy of urban areas over the total watershed area in the protection zones between these years.

**TABLE 2**
**Urban Area Changes in Protected Zones of Darlık Dam (2004–2006)**

| Area Prot. Zone | Area (ha) | 2006 URB (ha) | URB TWA (%) | 2005 URB (ha) | URB TWA (%) | 2004 URB (ha) | URB TWA (%) | 2006–2004 (%) | 2006–2005 (%) | 2005–2004 (%) |
|---|---|---|---|---|---|---|---|---|---|---|
| Total | 20,352.455 | 79.75 | 0.39 | 77.31 | 0.38 | 74.60 | 0.37 | 0.02 | 0.01 | 0.01 |
| Long | 15,024.915 | 70.81 | 0.47 | 68.22 | 0.45 | 63.22 | 0.42 | 0.05 | 0.02 | 0.03 |
| Medium | 1761.435 | 3.68 | 0.21 | 3.52 | 0.14 | 3.80 | 0.22 | –0.01 | 0.07 | –0.08 |
| Short | 1789.855 | 4.78 | 0.27 | 3.69 | 0.21 | 4.04 | 0.23 | 0.04 | 0.06 | –0.02 |
| Absolute | 1838.09 | 1.23 | 0.07 | 3.33 | 0.18 | 2.94 | 0.16 | –0.09 | –0.11 | 0.02 |

*Note:* Prot., protection; URB, Urban area; TWA, total watershed area.

## Multiple Regression Analysis

Digital number values of the Landsat TM satellite images were converted to unitless planetary reflectance values as described in Chandler et al.[26] to eliminate the effects of high local variability on RS observation values. This conversion is necessary for studies regarding reflectances of lake surfaces because raw digital numbers of a Landsat image are not only dependent on the reflectance characteristics of a specific scene, but also contain noise and digital number value offsets caused by the viewing geometry of the satellite, the angle of the sun's incoming radiation, the atmospheric depth due to viewing angle, and the design characteristics of the sensor.

After this conversion, atmospheric correction becomes necessary in order to obtain correct results because electromagnetic energy radiated by objects exhibits different properties under different atmospheric conditions dependent on the illumination and the geometrical shape of the object, mist, fog, and haze in the air. The objective of atmospheric correction is to retrieve the actual "clear sky" surface reflectance from remotely sensed imagery by removing the specific weather-related atmospheric noise from a specific scene. Atmospheric correction has been shown to improve the accuracy of image classification significantly in some instances, but decreases the accuracy in other instances[27]. The atmospheric correction was done using the Dark Object Subtraction (DOS) haze-reduction method[28]. Therefore, the minimum reflectance value in the infrared band, which was measured to be 0.019, was subtracted from the reflectance values in all bands. For this particular study area of lake surfaces, even this elementary operation improved the results obtained.

Multiple regression analyses were next carried out for each WQ parameter (chlorophyll-a [CHL], total deposited matter [TDM], Secchi disk, total phosphate, and total nitrate) measured at seven stations at satellite image acquisition date (Fig. 3, Table 3) to determine the unknown coefficients, a–g of model in Eq. 1, in order to calculate the WQ parameter at any location on the lake.

$$WQ = a + b_* x_1 + c_* x_2 + d_* x_3 + e_* x_4 + f_* x_5 + g_* x_7 \quad (1)$$

The unknown coefficients obtained at regression analyses are given in Table 4. This proves that the spectral bands of the Landsat satellite images are well capable of being used in WQ measurement studies. WQ maps created by these formulae are given in Fig. 4A–E.

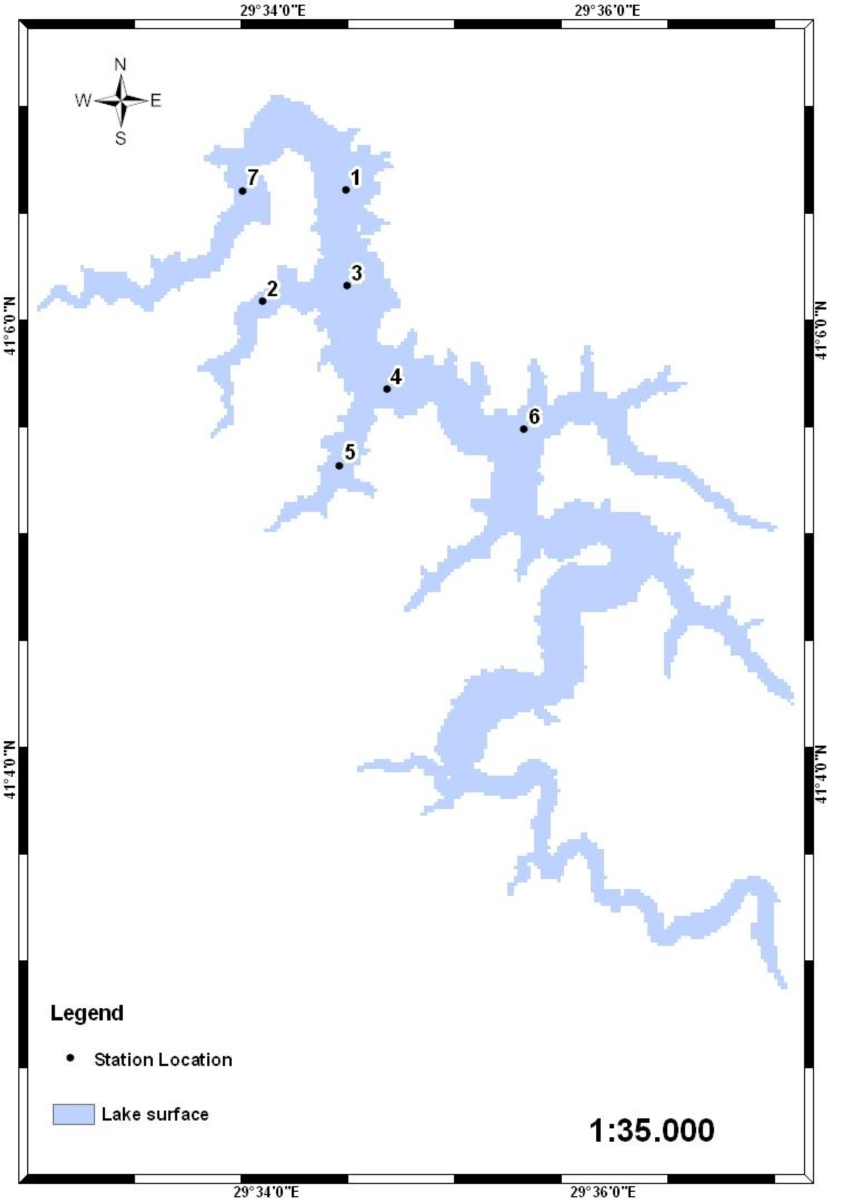

**FIGURE 3.** WQ measurement stations at Darlık Dam.

**TABLE 3**
**A. Atmospheric Corrected Landsat TM Unitless Planetary Reflectance Values at Bands 1–4**

| Parameter/ Station No. | TM Band 1 | TM Band 2 | TM Band 3 | TM Band 4 |
|---|---|---|---|---|
| 1 | 0.0855216 | 0.0640628 | 0.0436698 | 0.0366779 |
| 2 | 0.0784490 | 0.052957 | 0.0339565 | 0.0183389 |
| 3 | 0.0756200 | 0.052957 | 0.0339565 | 0.0244519 |
| 4 | 0.0841071 | 0.0611960 | 0.0611960 | 0.0412415 |
| 5 | 0.0657184 | 0.043955 | 0.0439955 | 0.0218149 |
| 6 | 0.0784490 | 0.0583292 | 0.0583202 | 0.0388132 |
| 7 | 0.0897651 | 0.0783966 | 0.0783966 | 0.0509548 |

**B. WQ Parameters Measured at the Stations***

| Parameter/ Station No. | CHL (mg/l) | Secchi Disk (m) | TDM (mg/l) | Total Phosphate (mg/l) | Total Nitrate (mg/l) |
|---|---|---|---|---|---|
| 1 | 8.37 | 3 | 207 | 0.043 | 0.302 |
| 2 | 2.04 | 3.5 | 206 | 0.039 | 0.241 |
| 3 | 2.91 | 2 | 207 | 0.045 | 0.258 |
| 4 | 3.86 | 2.3 | 210 | 0.046 | 0.279 |
| 5 | 8.95 | 1.5 | 211 | 0.049 | 0.346 |
| 6 | 7.25 | 1.8 | 211 | 0.046 | 0.286 |
| 7 | 5.29 | 3.3 | 205 | 0.049 | 0.289 |

* One measurement/station/satellite acquisition time[31,32].

**TABLE 4**
**Formulae Derived for Each WQ Parameter of Table 3B through Regression Analyses, for x, the Pixel Reflectance Values at Bands 1–4, using all the Data Available in Table 3**

| WQ Parameters | Formulae Derived |
|---|---|
| CHL (mg/m3) | 19.74-1953.17*x1+3224.205*x2-493.685*x3-905.316*x4 |
| Secchi disk (m) | -13.438+495.487*x1-347.095*x2-192.402*x3-125.433*x4 |
| TDM (mg/l) | -231.802-2201.22*x1+3212.838*x2-68.706*x3-1055.71*x4 |
| Total phosphate (mg/l) | 0.077-1.632*x1+2.046*x2-0.228*x3-0.412*x4 |
| Total nitrate (mg/l) | 0.185-17.322*x1+39.773*x2-13.77*x3-10.237*x4 |

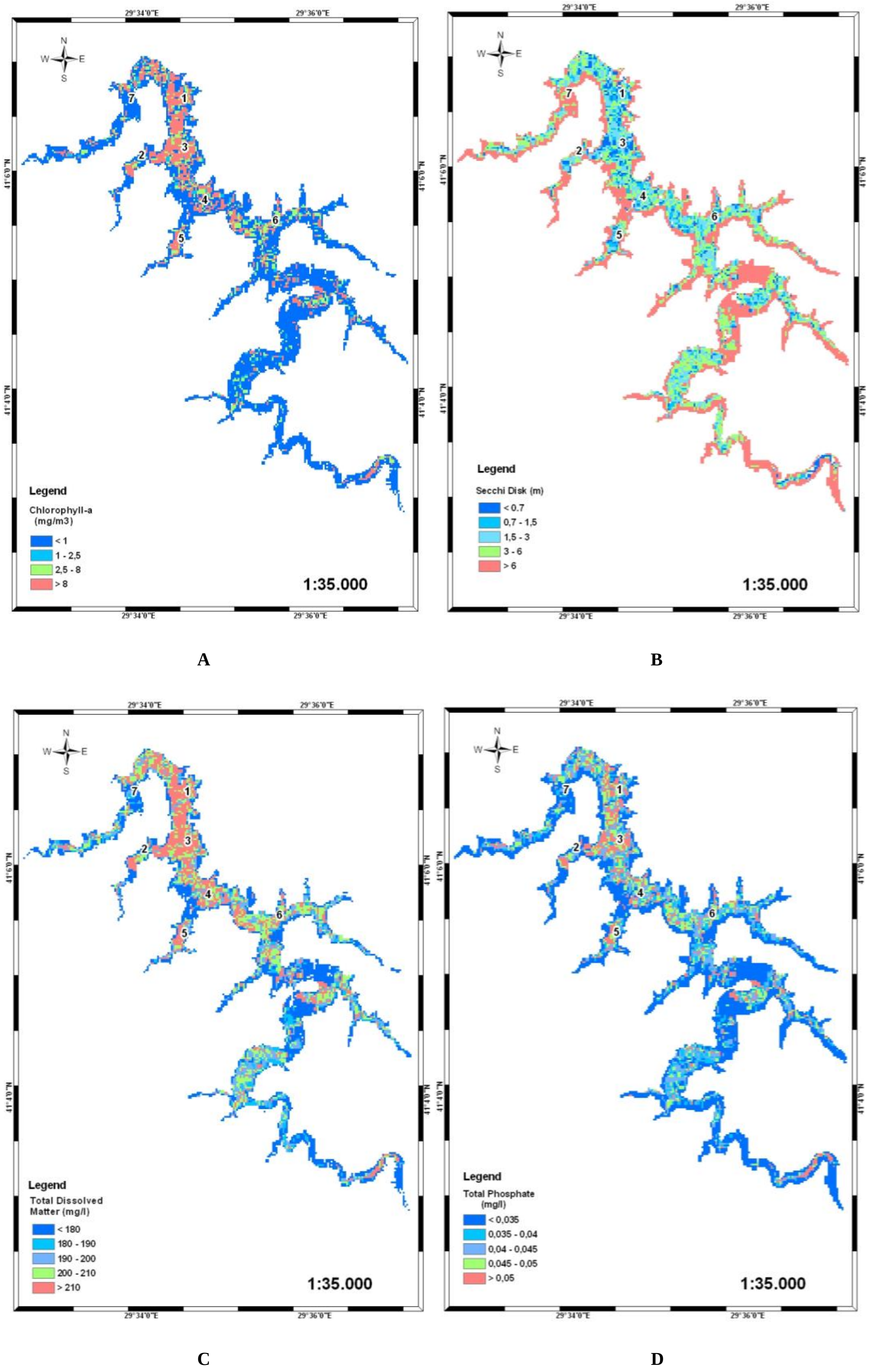

**FIGURE 4.** WQ images by regression analysis. (A) CHL, (B) Secchi disk, (C) TDM, (D) total phosphate, (E) total nitrate. *Continued next page.*

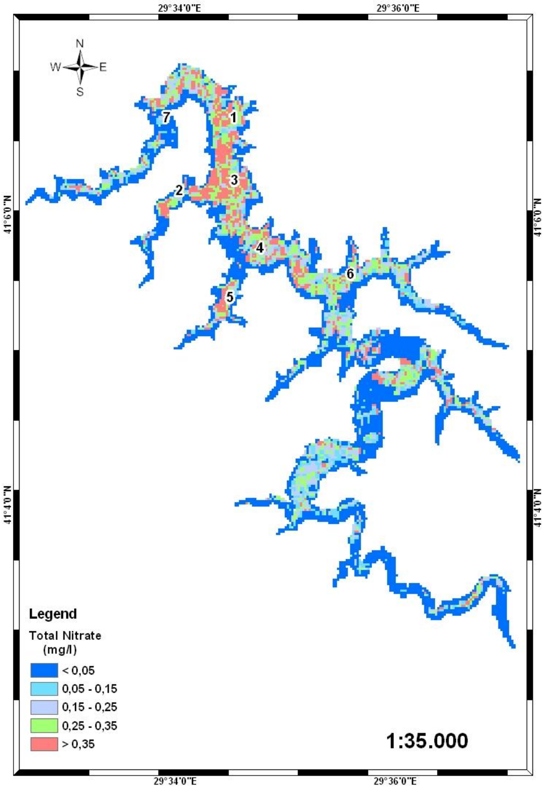

**FIGURE 4E**

## Multitemporal WQ Detection

Multitemporal WQ in Darlik Dam was investigated by pattern recognition techniques in this part of the study. The registered lake images corresponding to years 2004, 2005, and 2006 were classified by a supervised classification technique; namely, minimum distance classification[29]. The supervised classification results of Darlik Dam 2004, 2005, and 2006 Landsat 5 TM satellite images are shown in Fig. 5A–C.

Legends for WQ classification can be explained as

- Yellow: nutrient pollution–loaded agricultural wastewater
- Green: phosphorous pollution–loaded water which comes from forested areas
- Red: total suspended solids pollution–loaded, urban, agricultural, and industrial wastewater discharges
- Blue: natural lake water

Interpretation of the results reveals that all the point inlets from the rivers to the Darlik Dam carry slightly polluted water into the natural unpolluted lake water as shown with yellow, green, and red colors corresponding to different water qualities. Although urban areas are located in the long-range protection zone (LPZ) of the dam, domestic discharges may still be carried by rivers, and, particularly, the locations depicted in yellow or green show that agricultural activities increase to a minor extent in the vicinity of the arms of the rivers emptying into the dam. Nitrogen and phosphorus compound-enriched irrigation water remains from the agricultural areas and temporal changes in rivers carrying eroded soil are detected by processing satellite imagery of the area.

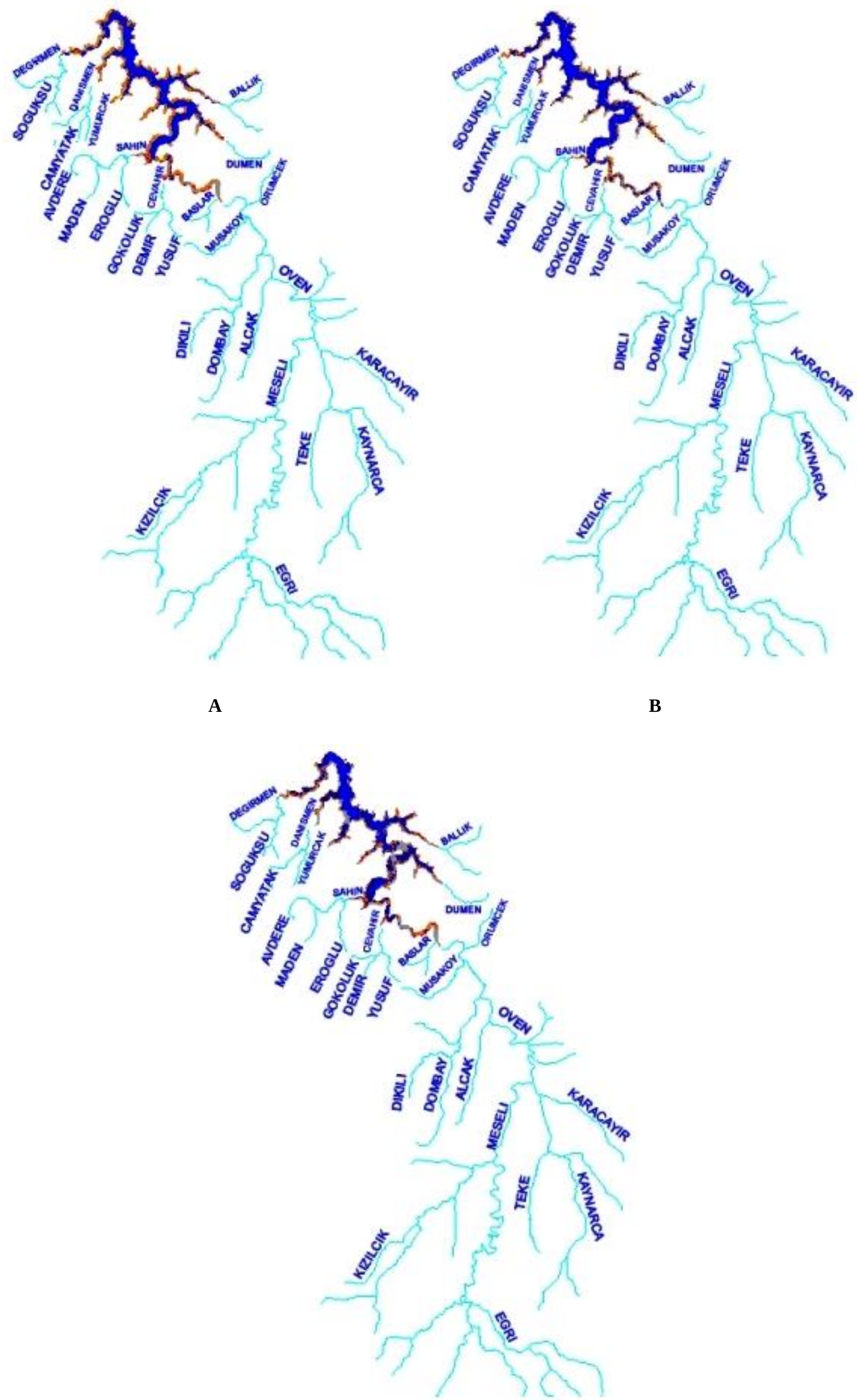

C

**FIGURE 5.** WQ images by pattern recognition techniques. (A) 2004, (B) 2005, (C) 2006.

WQ in 2004 was rather clean because of the rich water quantity in the month of December and the inappropriateness for the growth of microorganisms from a temperature point of view. In 2005 and 2006, WQ varied slightly depending on agriculture and forestry in the vicinity. Multitemporal WQ variation analysis at the Darlik Dam reveals that the dam has preserved its natural water and clean status (Table 1) since the year 2000.

## RESULTS AND DISCUSSION

Landsat 5 TM satellite images of the years 2004, 2005, and 2006 were used to determine land use/land cover changes in the Darlık Dam watershed. The analysis revealed that in 2006, urban areas covered at most 0.39% of the total watershed area, 89% of it being in the long-range protection zone (LPZ). The 2006 urban areas occupy only 1.23 ha of the absolute protection zone (APZ) and this amount shows a decrease compared to 3.33 ha of urban area of the APZ in 2005. Urban areas gradually increased between 2004 and 2006 by increments of 0.01%, which shows that the administrative authorities strictly controlled the area against illegal urbanization and maintained the potability of the Darlik Dam lake.

Nevertheless, slight pollution in the Darlık Dam originates from domestic wastewater discharged into the rivers that feed the lake in the LPZ, phosphorous and nitrogen compound-enriched irrigation water residues from agricultural areas in the hydrological watershed, and precipitation that carries eroded soil. The rivers in the watershed and those joining the dam are depicted in Darlik Dam WQ images for the purpose of emphasizing the effect of the rivers on the dam's WQ.

In this study, regression analysis was used to determine the WQ parameters of Darlık Lake, which is an indispensable potable water resource of Istanbul. *In situ* measurements at the satellite image acquisition date of the lake at seven stations at various parts of the lake, perfectly characterizing the WQ, were input into regression analysis for each WQ parameter, namely CHL, TDM, turbidity (Secchi disk), total phosphate, and total nitrate, which are considered as the significant ones per Bhavsar[30].

In the regression analyses, multiple R values were 0.93, 0.88, 0.92, 0.87, and 0.88 for CHL, Secchi disk, TDM, total phosphate, and total nitrate, respectively, which indicated that accurate maps will be obtained for the WQ parameters of Darlık Dam.

WQ maps of Darlik Dam shown in Fig. 4A–E reveal that CHL concentration in most of the lake is <8 $mg/m^3$, slightly increasing to the north of the lake; Secchi disk is between 1.5 and 3 m in most parts of the lake; TDM is between 180 and 210 mg/l in the entire lake; total phosphate concentration is mostly between 0.035 and 0.05 mg/l; and total nitrate concentration is between 0.25 and 0.35 mg/l in most parts of the lake These analyses show that Darlik Dam preserved its quality as a potable water resource of the year 2000 (also in years 2004, 2005, and 2006) and can be used with prescribed treatment.

This study may be considered as the first attempt of space technology applications for WQ monitoring and assessment. More years of data based on space images, particularly Landsat 5 TM, may be considered for land use/land cover issues. In the meantime, increasing the scale-studied area around the lake and the development of appropriate GIS data would be used more easily by authorities who highly need such information for further decision making.

## CONCLUSIONS

The objective of this study was to emphasize the potential for application of RS techniques and regression analyses for assessment of WQ, and investigation of the effectiveness of the implemented cleaning programs. Through the direct model established at the end of this study, it was possible to determine the WQ anywhere at the lake surface, feeding the measured WQ parameters at seven WQ measurement stations and Landsat 5 TM satellite image reflectances at these stations into the model. This study indicated that the implemented cleaning program is rather effective and the lake water is still potable with prescribed treatment. This is mainly because there is still negligible urbanization around the lake and no industry threatening this natural water resource. However, this situation may change in the future. Therefore, the lake WQ should be monitored at least once a month by the techniques introduced here in order to sustain its quality and still be able to take preventive measures in the protection zones of the lake when necessary. This study is original for Turkey, as it introduces for the first time the capability of satellite images for WQ assessment in a pilot study area, namely the Darlık Dam. The methodology employed for the Darlık Dam WQ analysis using RS and GIS analysis and multiregression techniques proved to be as successful as the one done for the Küçükçekmece Water Quality Analysis[1] because we could obtain WQ maps for the dam lake and investigate the multitemporal changes in its watershed area, and derive conclusions that will assist provincial administrators in their studies to preserve the WQ in the Darlık Dam. This research can be extended by using other complementary satellite data in the future.
